# Be Kind to Yourself: Testing Self-Compassion, Fear of Recurrence, and Generalized Anxiety in Women with Cancer Within a Multiple-Mediation Model

**DOI:** 10.3390/jcm14134696

**Published:** 2025-07-02

**Authors:** Dariusz Krok, Ewa Telka, Sebastian Binyamin Skalski-Bednarz

**Affiliations:** 1 Institute of Psychology, University of Opole, 45-040 Opole, Poland; 2 Department of Radiotherapy, Maria Sklodowska-Curie National Research Institute of Oncology, Gliwice Branch, 44-101 Gliwice, Poland; etelka@io.gliwice.pl; 3 Faculty of Philosophy and Education, Catholic University of Eichstätt-Ingolstadt, 85072 Eichstätt, Germany; sebastian.skalski@ku.de; 4 Institute of Psychology, Humanitas University, 41-200 Sosnowiec, Poland

**Keywords:** self-compassion, fear of recurrence, anxiety, psychological flexibility, coping, cancer

## Abstract

**Background/Objectives**: Self-compassion, characterized by treating oneself with kindness during challenges, has been shown to alleviate anxiety and fear of recurrence in cancer patients by mitigating negative emotions such as depression and fatigue. Psychological flexibility and coping strategies have emerged as potential mediators in the relationship between self-compassion and emotional well-being, suggesting that these factors play a critical role in managing distress in cancer patients. However, further research is needed to clarify the mechanisms through which self-compassion, psychological flexibility, and coping interact to influence fear of recurrence and anxiety in cancer populations. **Methods:** Two hundred and ninety-six women who had completed cancer treatment completed self-report assessments of self-compassion, psychological flexibility, coping, fear of recurrence, and generalized anxiety. Structural equation modeling (SEM) was applied to test a multiple-mediation model, including serial and parallel pathways among the study variables. **Results:** Self-compassion was positively associated with psychological flexibility and coping strategies, and negatively associated with fear of recurrence and generalized anxiety. Path analysis identified significant serial and parallel mediation effects, where both positive and negative self-compassion were associated with lower fear of recurrence and generalized anxiety through pathways involving psychological flexibility and emotion- and meaning-focused coping. The findings highlight the protective role of self-compassion in reducing psychological distress, suggesting that enhancing self-compassion may improve emotional adjustment in cancer patients. **Conclusions:** Attitudes based on self-kindness and a nonjudgmental approach have significant potential in reducing fear of cancer recurrence and generalized anxiety in female cancer patients, emphasizing the mediating role of psychological flexibility and coping strategies. According to the acceptance and commitment therapy (ACT) model, these relationships highlight the important roles of personal resources and coping mechanisms in alleviating negative emotional states in women with cancer. Interventions focused on self-compassion and psychological flexibility could provide valuable support in coping with the emotional suffering associated with cancer.

## 1. Introduction

Self-compassion, which reflects treating oneself with kindness and consideration in the face of challenges and difficulties, has promoted emotional well-being in various populations, including those facing chronic health conditions such as cancer [1,2]. For cancer patients, self-compassion can serve as an important psychological resource, helping to alleviate fear and anxiety related to the illness. Identifying the factors and mechanisms that mediate the relationships between self-compassion, fear of recurrence, and anxiety is thus of critical importance for both scientific inquiry and clinical practice.

### 1.1. The Role of Self-Compassion in Mitigating Negative Emotions in People with Cancer

Self-compassion, as defined by Neff [3], refers to an attitude of kindness and understanding toward one’s inadequacies and failures, rather than being harshly self-critical. It fosters a nonjudgmental stance toward personal suffering and difficulties (e.g., chronic illness), situating such experiences within the broader context of human existence. Self-compassion can be divided into two forms [4]: (1) positive self-compassion, which refers to the ability to respond to one’s suffering with kindness, understanding, and a sense of shared human experience, and (2) negative self-compassion, which involves maladaptive responses based on self-judgment, isolation, and over-identification. Although they are not complete opposites, as a moderate positive correlation still exists between them, these dimensions may function differently and have distinct psychological correlates with various indicators of mental health [5].

Recent studies suggest that self-compassion may be a protective factor in mitigating the negative effects of fear and anxiety in cancer. In the context of cancer, patients often face intense psychological challenges, including fear of recurrence or uncertainty about the future related to health, mental changes, or treatment issues. Self-compassion may thus help patients regulate these emotional responses more effectively. For example, rather than becoming overwhelmed by fear or engaging in harsh self-criticism, a self-compassionate individual is more likely to acknowledge their fears without judgment, respond with kindness, and maintain a balanced perspective. Research showed that in patients diagnosed with cancer for the first time, a self-compassion total score, negative self-compassion, and, to a lesser extent, positive self-compassion were negatively associated with anxiety, depression, and fatigue around cancer diagnosis. In addition, throughout treatment, positive self-compassion was more strongly associated with the trajectory of symptoms of depression, anxiety, and fatigue over time compared to negative self-compassion [6]. Among breast cancer survivors, self-compassion was found to be negatively related to anxiety, depression, and fear of dying [7]. Patients with heterogeneous cancer types, who were characterized by high levels of self-compassion, also reported fewer symptoms of depression and anxiety, indicating a mitigating role of self-compassion in the experience of negative emotional states [1].

These results demonstrate that self-compassion helps individuals to attenuate anxiety and maintain a balanced attitude in the face of stressful cancer situations. It enables patients to respond to fear and anxiety with kindness and acceptance rather than self-criticism or avoidance. As a consequence, this reassuring attitude helps regulate distress and foster adaptive coping, thereby mitigating the negative psychological impact of cancer-related challenges.

Although self-compassion is generally regarded as a protective factor, research directly examining its relationship with fear of recurrence among cancer patients remains relatively limited, which—once treatment is finished—occurs in around 70% of patients [8]. Furthermore, existing studies highlight its complexity, suggesting that this association may be complex and not entirely straightforward. Research on the effectiveness of self-compassion-based interventions among cancer patients has demonstrated that employing techniques grounded in self-compassionate attitudes is associated with a reduction in fear of cancer recurrence in both breast cancer survivors [9,10] and a general sample of adult cancer patients [11]. These interventions using self-compassion facilitated patients’ activation of motivational, cognitive, and emotional resources, as well as enabled them to cope constructively with the anxiety triggered by thoughts, images, or memories associated with fear of cancer recurrence. Among women with breast cancer who had completed treatment, higher levels of self-compassion were found to be negatively associated with worry and ruminative thinking—core cognitive features underlying the fear of cancer recurrence [12]. This is further supported by Wei et al. [13], who developed and tested a fear-focused self-compassion therapy tailored for young breast cancer patients in China. Their randomized controlled trial aimed to reduce severe fear of recurrence by enhancing self-compassion and modifying maladaptive cognitive patterns such as catastrophizing and rumination. This suggests that self-compassion may help mitigate maladaptive cognitive patterns that contribute to ongoing distress in cancer experiences.

Recent systematic reviews and meta-analysis on self-compassion-based interventions and fear of recurrence in oncology have yielded interesting and valuable conclusions. The meta-analysis conducted by Fan et al. [14] demonstrated that compassion-based interventions significantly reduced depression and enhanced self-compassion. Moderation analyses also revealed that structured, facilitator-led interventions produced greater improvements in self-compassion compared to brief formats. Treating themselves with compassion tends to enhance emotional regulation and protect cancer patients from psychopathological symptoms. In their examination of self-compassion-based interventions in oncology, Grégoire et al. [15] found that all types of interventions (e.g., Compassion-Focused Therapy (CFT), self-compassion exercises, and Mindful Self-Compassion (MSC) training) led to improvements in cancer patients’ ability to treat themselves kindly. Most of these interventions were also associated with reductions in anxiety, depression, fatigue, and pain. Additionally, several studies reported positive associations with treatment adherence and quality of life. Having reviewed randomized controlled trials on the efficacy of compassion-based interventions in breast cancer patients, López-Contreras et al. [16] concluded that all interventions led to significant increases in self-compassion and acting with awareness, as well as significant reductions in anxiety, stress, depression, and negative affect. These findings provide evidence for the clinical utility of self-compassion in helping cancer patients cultivate a compassionate attitude following traumatic experiences, thereby promoting emotional regulation and alleviating negative affective states within the oncological context.

However, some studies have proven to be inconclusive, yielding ambiguous results. In a cross-sectional study of women with breast cancer, it was found that while the total score of self-compassion was negatively associated with fear of recurrence, negative self-compassion was positively associated with this fear, and positive self-compassion did not show a significant association [17]. However, in patients with heterogeneous types of cancer, total self-compassion as well as positive and negative self-compassion were negatively related to anxiety symptoms, which are inherent in fear of recurrence. In addition, the relationship between self-compassion and anxiety was mediated by other psychological factors: patients’ perceptions of the consequences of cancer and the belief in a cyclical timeline of the illness [18]. This suggests that the associations between self-compassion and both anxiety and fear of recurrence are likely influenced by underlying psychological factors and processes that may determine the direction of these relationships. However, further research is warranted to elucidate the potential mediating mechanisms.

### 1.2. Psychological Flexibility and Coping as Potential Mediators

Empirical evidence indicates that the relationship of self-compassion with anxiety and fear of recurrence may be mediated by additional psychological factors, such as psychological flexibility and coping strategies. In a group of patients with heterogeneous cancer types, psychological flexibility was found to mediate the relationship between self-compassion and posttraumatic growth [19], underscoring the potential of this mental resilience factor as a significant protective mechanism in cancer. In post-treatment cancer patients, psychological flexibility was a serial mediator along with mentalization between cancer-related total pain and fear of recurrence [20]. Being a resilient factor in mental health, psychological flexibility also served as a mediator between cognitive emotion regulation mechanisms and subjective well-being in patients with breast cancer [21]. These results imply that psychological flexibility that reflects personal capacities to confront challenging internal and external experiences, as well as to interpret one’s behavior through a motivational lens, can mitigate the negative emotions and psychological distress associated with cancer.

The mediating role of psychological flexibility for anxiety and fear outcomes was also found in non-clinical samples. Among university students, psychological flexibility mediated the relationship between traumatic memories of shame and symptoms of anxiety and depression [22]. Specifically, traumatic memories of shame were negatively related to psychological flexibility, which in turn was negatively related to anxiety and depression. Psychological inflexibility (the reverse of psychological flexibility) was also found to mediate the effects of a mindfulness-based intervention, which considerably relied on self-compassion techniques, on depression and stress symptoms in a sample of oncology nurses [23].

These positive effects of psychological flexibility may be due to its capacity to facilitate coping processes in the context of cancer by enabling more adaptive and coherent interpretations of the illness experience [21,24]. The functional mechanisms underlying the association of psychological flexibility and coping with anxiety and fear can be explained within the acceptance and commitment therapy (ACT) framework [25,26]. According to ACT, aversive internal experiences resulting from serious illness, such as distressing cognitions, emotions, and physical sensations, are inherent aspects of human life. However, psychological flexibility—the ability to stay in contact with the present moment and act in accordance with personal values despite unpleasant thoughts or feelings—allows individuals to acknowledge and accept difficult internal experiences (e.g., fear, worry) without attempting to control or avoid them [26]. This allows individuals to respond to anxiety with more effective coping mechanisms, such as acceptance and value-driven action, rather than through maladaptive avoidance, which tends to sustain or intensify anxiety over time.

The model posits that when individuals are psychologically flexible, they are more likely to engage in adaptive coping strategies, such as meaning-focused coping or problem-solving, rather than avoidance-based strategies. This helps reduce the intensity and frequency of anxiety and fear in the face of illness or uncertainty. In the context of cancer, self-compassion may reduce self-judgment and emotional avoidance, creating a basis for more open, accepting engagement with difficult emotions like fear and anxiety [6,10]. This openness can strengthen psychological flexibility, which will allow individuals to face cancer-related fears (such as fear of recurrence) without being overwhelmed or controlled by them. In turn, higher psychological flexibility can lead to lower levels of distress, including reduced generalized anxiety and less intrusive fear of recurrence.

The aforementioned assumptions of the model are supported by empirical findings. In heterogeneous cancer patients, active coping strategies (i.e., self-confident approach, optimistic approach, and seeking social support) were mediators in the association between psychological flexibility and posttraumatic growth [27]. Furthermore, three coping strategies: confrontation, avoidance, and acceptance-resignation mediated the relationship of self-compassion with body image disturbance in young breast cancer survivors [28]. Mediational effects were also found in non-clinical samples. Two coping strategies, positive reframing and acceptance, mediated the relationship of positive self-compassion, but not negative self-compassion, with posttraumatic growth in college students. Specifically, positive self-compassion was positively associated with the coping strategies, which, in turn, were linked to higher levels of posttraumatic growth [29]. Psychological flexibility and approach and avoidant coping styles were serial mediators in the pathway between experiencing stressful events (i.e., COVID-19) and psychological distress in the Australian population [30].

Although previous studies have identified modest associations among the above variables, to the best of our knowledge, none have examined psychological flexibility and coping strategies within a multiple-mediation model—incorporating both serial and parallel effects—in the relationship between self-compassion and fear of recurrence and anxiety among women with cancer. Given the inclusion of non-clinical samples and the use of heterogeneous cancer samples, it remains unclear to what extent the conclusions can be generalized to the female cancer population [13,18,21]. By focusing specifically on a female clinical population and employing a path analysis approach, the present study seeks to address this gap and provide a more refined understanding of the psychological mechanisms that may buffer emotional distress in the context of oncological illness.

### 1.3. The Present Study

This study aimed to examine whether psychological flexibility and coping would mediate the relationship of self-compassion with fear of recurrence and generalized anxiety in women with cancer. Based on the ACT theory and previous research [17,22,27], we hypothesized that self-compassion would be negatively associated with fear of recurrence and generalized anxiety, but positively associated with psychological flexibility and coping. Next, psychological flexibility would mediate the effect of self-compassion on fear of recurrence and generalized anxiety. Finally, psychological flexibility and coping would serially and parallelly mediate the effect of self-compassion on fear of recurrence and generalized anxiety.

## 2. Materials and Methods

### 2.1. Participants

Two hundred and ninety-six women previously diagnosed with cancer participated in this study. The participants were diagnosed with different types of cancer (i.e., gastrointestinal, breast, uterine, skin, and lung) and underwent radical cancer treatment (i.e., radiotherapy, chemotherapy, targeted therapy, immunotherapy, and hormone therapy) in oncological centers. As a consequence, they were in disease remission, which indicates that there were no detectable signs of active cancer at the time of assessment. Their age ranged from 26 to 86 years (M = 54.68; SD = 13.71). With regard to educational levels, 11 women had primary education (3.7%), 64 had basic vocational education (21.6%), 83 had secondary education (28.1%), and 138 had higher education (46.6%). Inclusion criteria were as follows: (1) confirmed diagnosis of cancer (stages from I to III), (2) cognitive capacity to fill in questionnaires, (3) lack of other medical conditions that could affect the patient’s responses (e.g., stage IV cancer, heart disease), (4) positive reactions to treatment, and (5) having successfully completed treatment and being in remission.

### 2.2. Procedure

Participants were recruited from oncological units in the southern parts of Poland between February 2024 and January 2025 while undergoing medical treatment. They were personally approached by oncologists who examined their medical conditions in terms of the above-mentioned study criteria. Once they were met, the suitable patients were invited to participate in the study and informed about its procedure. Informed consent was acquired from all participants. Initially, 334 patients were invited to participate, of whom 24 declined to participate, and 14 had medical conditions that excluded them from participation; thus, the final number was 296 participants (88.62%). This study was conducted in a flexible manner to accommodate participants’ preferences and timetables. Specifically, data collection took place either in clinical settings immediately following patients’ medical appointments, or participants were given the option to take the questionnaires home and complete them later, depending on what was most convenient for each individual. Participation was entirely voluntary and anonymous, with informed consent obtained from all participants before their involvement.

### 2.3. Measures

Self-compassion. The 26-item Self-Compassion Scale (SCS) was used to measure self-compassion [31]. It comprises six components: self-kindness, self-judgment, common humanity, isolation, mindfulness, and over-identification. Although the original model of self-compassion by Neff [3,31] conceptualizes it as a unified construct with interrelated subscales, recent studies [32,33,34] have supported a two-factor structure distinguishing between positive and negative dimensions. In line with this framework, we adopted the positive–negative self-compassion distinction in the current study. Additionally, the overall score was calculated as the arithmetic mean of the positive and negative dimensions. Participants assessed items using a scale, ranging from 1 (almost never) to 5 (almost always); a higher score indicated either more positive or more negative self-compassion. In this study, the Cronbach’s coefficients for positive and negative self-compassion were 0.89 and 0.87, respectively.

Psychological flexibility. The 7-item Acceptance and Action Questionnaire was employed to assess participants’ psychological flexibility, defined as the capacity to stay fully engaged with the present moment and to pursue value-driven goals, even in the face of challenging internal and external experiences or obstacles [35]. Items were scored by using a scale, ranging from 1 (never true) to 7 (always true). A higher score indicated a greater degree of psychological flexibility. In the current study, the Cronbach’s coefficient was 0.89.

Coping. The 37-item Coping Questionnaire was applied to measure three coping strategies: problem-focused, emotion-focused, and meaning-focused [36]. Together, the subscales capture how individuals routinely draw on these strategies across various life domains, providing a comprehensive profile of their coping style in response to stress. Items were assessed by using a scale, ranging from 1 (not at all) to 5 (very much). A higher score indicated greater use of the coping strategy. In this study, the Cronbach’s coefficients for problem-focused, emotion-focused, and meaning-focused strategies were 0.84, 0.85, and 0.87, respectively.

Fear of recurrence. The extended 8-item Cancer Worry Scale was utilized to evaluate concerns regarding the potential recurrence of cancer and the impact of these concerns on daily functioning [37]. Items were scored using a scale, ranging from 1 (never) to 4 (almost always). As the scale assesses fear or worry related to the recurrence or progression of the disease within the same organ or to other parts of the body, it can be applied to both cancer survivors and patients with active (i.e., non-remittent) cancer [38]. A higher score reflected greater fear of cancer recurrence. In the current study, the Cronbach’s coefficient was 0.85.

Generalized anxiety. The 7-item Generalized Anxiety Disorder (GAD-7) questionnaire was used to measure participants’ overall level of anxiety [39]. It assesses the extent to which the patient has experienced difficulties such as feeling anxious, not being able to stop or control worrying, being restless and easily irritable, and feeling afraid of something unexpected during the previous 2 weeks. Items were assessed on a scale, ranging from 1 (not at all) to 4 (nearly every day). Higher scores indicate higher anxiety. In the current study, the Cronbach’s coefficient was 0.88.

### 2.4. Data Analysis

Preliminary calculations, based on earlier studies and power estimations, indicated that a minimum of 263 participants would ensure adequate statistical power (calculated using the method by Faul et al. [40], for a sample size required to achieve 80% power (1 − β), with α = 0.05 and an effect size of 0.05). With regard to specific parameters in conducting power analysis, we applied the following type of analysis: *F* test; linear multiple regression: fixed model, R2 deviation from zero; and a priori-type power analysis. To account for gender-stratified analyses, we slightly increased the sample size to *N* = 296. To exclude potential biases due to common method variance, we conducted Harman’s one-factor test [41]. The results showed that common method bias was not a concern in this study (18 distinct factors, with the first factor explaining only 23.85% of the variance). Additionally, to minimize the risk of Type II errors, we applied a bootstrapping procedure with 5000 samples and 95% confidence intervals.

To provide an initial examination of associations among self-compassion, psychological flexibility, coping, fear of recurrence, and generalized anxiety, a bivariate correlation analysis was conducted. To further investigate the hypothesis that the relationship of self-compassion with fear of recurrence and generalized anxiety is serially mediated through psychological flexibility and coping, path analysis was selected as the analytic framework, as it permits the simultaneous estimation of multiple indirect effects while accounting for the unique variance attributed to each mediator. Path analysis was conducted within the structural equation modeling (SEM) framework using the maximum likelihood (ML) estimation method. Analyses were conducted using the AMOS 26 software, with 5000 bootstrap samples and 95% bias-corrected confidence intervals to estimate both direct and indirect effects in single and multiple mediator models. These parameters ensured accurate statistical analyses by providing appropriate control over potential confounding variables and enhancing the reliability of the results.

Model fit was evaluated using multiple indices: the chi-square-to-degrees of freedom ratio (*χ*^2^/df), the comparative fit index (CFI), the Tucker–Lewis index (TLI), the root mean square error of approximation (RMSEA), and the standardized root mean square residual (SRMR). Values of *χ*^2^/df < 3, CFI and TLI ≥ 0.90, RMSEA < 0.08, and SRMR < 0.08 were interpreted as indicators of acceptable model fit [42].

## 3. Results

### 3.1. Descriptive Statistics and Preliminary Analysis

Table 1 presents the means, standard deviations, and Pearson correlation coefficients for all study variables.

As hypothesized, the total score of self-compassion was significantly and positively associated with psychological flexibility, problem-focused coping, emotion-focused coping, and meaning-focused coping, whereas it was negatively associated with fear of recurrence and generalized anxiety. Positive self-compassion was positively correlated with psychological flexibility, problem-focused coping, emotion-focused coping, and meaning-focused coping. Negative self-compassion also showed significant positive correlations with psychological flexibility and all three coping strategies: problem-focused, emotion-focused, and meaning-focused. Moreover, positive and negative self-compassion were negatively associated with fear of recurrence and generalized anxiety, suggesting a protective role in psychological adjustment.

Psychological flexibility was positively associated with all coping strategies and negatively associated with both fear of recurrence and generalized anxiety, highlighting its role as a core resilience factor. The three coping strategies were all negatively correlated with both fear of recurrence and generalized anxiety. As expected, fear of recurrence and generalized anxiety were strongly and positively associated, underscoring the co-occurrence of these indicators of psychological distress.

### 3.2. Multiple Mediation Effects

**Figure 1:**
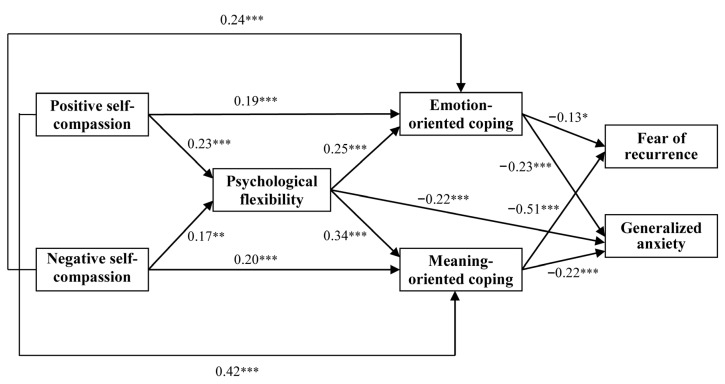
The final path analysis model between self-compassion, and fear of cancer recurrence and generalized anxiety. * *p* < 0.05, ** *p* < 0.01, *** *p* < 0.001.

Multiple mediation effects were examined by using path analysis in SEM. The initial model, including directional paths among self-compassion, psychological flexibility, coping, fear of recurrence, and generalized anxiety, was tested. However, the model with four mediators (psychological flexibility, problem-focused, emotion-focused, and meaning-focused) had an unsatisfactory fit to the data: *χ*^2^(39) = 151.41, *p* < 0.001; NFI = 0.83; IFI = 0.83; CFI = 0.84; RMSEA = 0.31; SRMR = 0.13; Hoelter’s index = 30. Several paths in the initial model were found to be statistically nonsignificant (*p* > 0.05). Following retesting guidelines, the model was re-specified to improve fit with some direct and indirect paths being removed. The revised model demonstrated a substantial and statistically significant improvement in fit: *χ*^2^(5) = 15.28, *p* < 0.001; NFI = 0.97; IFI = 0.98; CFI = 0.98; RMSEA = 0.07; SRMR = 0.03; Hoelter’s index = 293. All fit indices exceeded conventional thresholds for acceptable or good model fit, suggesting that the revised model was more accurate for the underlying relationships among the study variables (Figure 1). Furthermore, a chi-square difference test revealed that the final model was significantly better than the initial model, Δ*χ*^2^(5) = 136.13, *p* < 0.001. All indices indicated that the final model provided an optimal representation of the data. The final model includes the following seven variables: positive self-compassion, negative self-compassion (independent variables), psychological flexibility, emotion-oriented coping, meaning-oriented coping (mediators), fear of recurrence, and generalized anxiety (dependent variables).

No direct relationships were observed between positive self-compassion, negative self-compassion, and fear of recurrence or generalized anxiety. There were only indirect relationships among variables, which indicates the occurrence of mediational effects. To investigate these hypothesized mediational pathways, a bootstrapping approach was employed, offering a robust estimation of indirect effects. The analyses revealed four significant serial and parallel mediation effects (Table 2). The first two serial and parallel mediation models concerned positive self-compassion as the independent variable.

In the first mediation model, positive self-compassion was associated with lower levels of fear of recurrence through a sequential pathway involving psychological flexibility (Mediator 1), and emotion-oriented coping and meaning-oriented coping (Mediators 2). Considering the signs of the relationships between the individual variables, it can be concluded that higher levels of positive self-compassion were associated with greater psychological flexibility, which, in turn, was related to higher levels of emotion-focused and meaning-focused coping, ultimately leading to lower fear of recurrence. In the second mediation model, positive self-compassion was related to lower levels of generalized anxiety through a sequential pathway involving psychological flexibility (Mediator 1) and both emotion-focused and meaning-focused coping (Mediators 2). Higher positive self-compassion was associated with greater psychological flexibility, which, in turn, was related to higher levels of emotion-focused and meaning-focused coping, ultimately leading to lower levels of generalized anxiety. A comparison of standardized coefficients indicated a stronger mediation effect for fear of recurrence (E = −0.28; *p* < 0.001) than for generalized anxiety (E = −0.21; *p* < 0.001).

The next two serial and parallel mediation models included negative self-compassion as the independent variable. In the third mediation model, negative self-compassion was related to lower fear of recurrence through a sequential pathway involving psychological flexibility (Mediator 1) and both emotion-focused and meaning-focused coping (Mediators 2). Higher levels of negative self-compassion were associated with increased psychological flexibility, which, in turn, was linked to greater use of emotion-focused and meaning-focused coping, ultimately resulting in reduced fear of recurrence. Finally, the fourth model indicated that negative self-compassion relates to lower generalized anxiety through a sequential pathway involving psychological flexibility (Mediator 1), as well as emotion-focused and meaning-focused coping (Mediators 2). Higher levels of positive self-compassion were associated with increased psychological flexibility, which, in turn, facilitated greater use of emotion-focused and meaning-focused coping, eventually contributing to lower levels of generalized anxiety. A comparison of standardized coefficients revealed a similar mediation effect between fear of recurrence (E = −0.17; *p* < 0.001) and generalized anxiety (E = −0.16; *p* < 0.001). The similarity reflects that fear of recurrence and generalized anxiety may be affected by similar underlying psychological processes, particularly those related to psychological flexibility and coping strategies. This may imply that fear of recurrence and generalized anxiety are related or arise from similar experiences (e.g., maladaptive responses based on self-judgment, isolation, and over-identification).

In addition to the above serial and parallel mediation models, simple mediating effects were also observed. Psychological flexibility was a single mediator between positive and negative self-compassion and generalized anxiety, respectively. Emotion- and meaning-oriented coping were also parallel mediators between both positive and negative self-compassion, and fear of recurrence and generalized anxiety, respectively.

Given the cross-sectional design of the current study, alternative mediation models were tested to evaluate the robustness and directionality of the hypothesized associations. Specifically, a reversed order of mediators—placing coping strategies as the first mediators and psychological flexibility as the second—was examined. However, this alternative model demonstrated a poorer fit to the data: *χ*^2^(5) = 15.28, p < 0.001; NFI = 0.88; IFI = 0.88; CFI = 0.87; RMSEA = 0.23; SRMR = 0.07; Hoelter’s index = 54. Moreover, the indirect effect of emotion- and meaning-oriented coping, followed by psychological flexibility, on the relationship between self-compassion and fear of recurrence was not supported.

## 4. Discussion

Self-compassion has previously been shown to play a significant role in fear of recurrence and generalized anxiety in cancer patients [1,7]; yet, the underlying mechanisms—including the mediating role of psychological flexibility and coping strategies—remain poorly understood. In line with the broader literature, our study identified a multiple-mediation model involving psychological flexibility, emotion-focused coping, and meaning-oriented coping as mediators. These findings suggest that the factors of mental resilience (psychological flexibility) and personal skills (coping) examined in our study may regulate the negative emotional experiences of women with cancer.

### 4.1. Relationships Among Self-Compassion, Fear of Recurrence, and Generalized Anxiety

Consistent with previous findings on the association between self-compassion and anxiety and depression in different groups of cancer patients [6,11,12], we found that higher levels of total self-compassion as well as positive and negative self-compassion were associated with lower fear of recurrence and generalized anxiety. Self-compassion, the practice of offering oneself kindness and understanding during times of suffering, was shown to have a positive association with emotional well-being among women with cancer [6], especially in challenging situations such as cancer treatment and recovery. Our study has broaden this view by demonstrating that for female cancer patients, self-compassion can serve as an important psychological resource, helping to enhance resilience and decrease fear of recurrence in the face of illness. However, our findings do not align with those of Zhu et al. [17], who reported a positive association between negative self-compassion and fear of recurrence, while finding no significant relationship for positive self-compassion. One possible explanation for this discrepancy lies in the differences between study populations. Whereas Zhu et al.’s [17] research focused exclusively on women with breast cancer, our sample comprised women diagnosed with various types of cancer. Given recent findings that emotional responses to cancer may vary depending on cancer type and illness trajectory [43], these differences in sample characteristics may have contributed to the divergent results.

Fear of recurrence, a common experience among cancer patients, refers to the persistent worry that cancer may return after treatment [44]. This frequently triggers heightened levels of anxiety, further complicating the emotional challenges cancer patients face. Drawing on our findings, we conclude that female cancer patients who practice self-compassion are more likely to approach their fears with greater emotional flexibility, which may buffer the influence of anxiety and reduce the psychological burden associated with the uncertainty of cancer recurrence. In addition, our results showed that both forms of self-compassion contributed to lower generalized anxiety, with each effect remaining significant while controlling for the other. This suggests that both the attitude of positive self-compassion—based on the ability to respond to one’s suffering with kindness, understanding, and a sense of shared human experience—and the attitude of negative self-compassion—expressed through harsh self-criticism, isolation, or over-identification—help in coping with the burden of cancer and reduce negative emotional responses such as fear and anxiety. Such a relationship indicates the compatibility of both forms of self-compassion in influencing emotions, as suggested by earlier studies as well [5,6].

This result is in line with previous studies on breast cancer survivors [7] and patients with heterogeneous cancer types [1], in which having an attitude of kindness and understanding toward one’s inadequacies and failures buffered feelings of uneasiness and worry. As among female cancer patients, the psychological toll of diagnosis, treatment, and the uncertainty of recurrence often contributes to elevated levels of generalized anxiety [10,12], self-compassion may function as a protective factor against emotional distress, possibly due to its role in enhancing emotional regulation and reducing maladaptive self-criticism. Understanding this relationship helps to develop supportive interventions aimed at improving self-kindness and nonjudgmental attitudes in women coping with cancer.

### 4.2. The Serial and Parallel Mediation Effects

The primary finding of our study is that the relationship of positive and negative dimensions of self-compassion with fear of recurrence and generalized anxiety is mediated through both serial and parallel pathways involving psychological flexibility as well as emotion- and meaning-oriented coping strategies. Specifically, women who demonstrate greater kindness and understanding toward their own inadequacies and failures are more likely to exhibit psychological flexibility, enabling them to remain present-focused and committed to long-term values. This, in turn, fosters greater reliance on adaptive emotional and meaning-based coping strategies, which ultimately contribute to reduced fear and anxiety related to potential cancer recurrence.

In line with previous research showing the predominantly indirect relationships between self-compassion and negative emotions among cancer patients [18,28], our findings emphasize the dynamic interplay of personal resources (psychological flexibility) and coping strategies (emotion- and meaning-oriented coping) as potential mechanisms mediating the association between self-compassion and fear of recurrence and generalized anxiety in female cancer patients. However, the present study extends previous findings by demonstrating that the mediating mechanisms involve both positive and negative components of self-compassion. Specifically, higher levels of both positive and negative self-compassion were associated with increased psychological flexibility (serial mediator), which, in turn, was linked to greater use of emotion-focused and meaning-focused coping strategies (serial and parallel mediators), ultimately resulting in reduced fear of cancer recurrence.

This finding is particularly valuable and captivating, as it indicates ‘a functional cooperation’ between psychological flexibility and coping. Although our study precludes definitive conclusions about the direction of causality, the sequential order presented—psychological flexibility as the first mediator and coping strategies as the second—appears logical and justified. Acting as underlying mediating factors, psychological flexibility allows cancer patients to use both adaptive emotion-focused coping strategies, such as emotional expression or seeking social support, and meaning-focused ones, such as finding purpose or positive significance in the cancer experience [27,45] to effectively deal with illness. In this sense, psychological flexibility acts as a metacognitive foundation that empowers cancer patients to confront emotional challenges and cultivate meaning, thereby enhancing psychological adaptation and reducing fear of recurrence and anxiety. First, it can enable cancer patients to develop a broader awareness of their thoughts and emotions without becoming entangled or overwhelmed by them. Patients will be able to observe their internal experience, such as fear, anxiety, or distress, from a conscious and attentive perspective, rather than reacting impulsively or engaging in avoidance. Second, psychological flexibility can empower patients to confront emotional challenges directly, accepting difficult feelings as part of their lived experience instead of resisting or suppressing them.

This interpretation finds conceptual support in the theoretical framework of ACT, which posits that psychological flexibility is central to psychological well-being. According to the ACT model, self-compassion could facilitate female cancer patients’ psychological flexibility by endorsing a nonjudgmental stance toward internal experiences and reducing experiential avoidance. This adaptive mindset would enable women with cancer to engage more fully with their emotions and construct meaning from their experiences, rather than being dominated by fear or avoidance. The observed mediational pathway also suggests that self-compassion could support cognitive processing and emotional regulation in the face of cancer-related distress, particularly through mechanisms emphasized in ACT, such as mindfulness, acceptance, and values-based action. Consequently, it would be likely to alleviate fear of recurrence and generalized anxiety.

Additionally, the absence of problem-focused coping in our model may suggest that women, after successful treatment, are less oriented toward taking concrete actions or implementing strategies aimed at directly resolving illness-related issues. This may be attributable to the fact that, following the completion of treatment and the achievement of remission, there is no longer a perceived need for active problem-solving related to medical intervention [46,47]. Instead, reliance on emotion-focused and meaning-focused coping strategies may foster greater emotional awareness and self-reflection, which can, in turn, encourage adaptive psychological adjustment and constructive engagement with one’s post-treatment experience. It will help patients to distance themselves from a range of treatment-related activities that previously generated a significant mental burden.

The above findings also point to a growing need to integrate self-compassion-based strategies into psychosocial oncology interventions, given the accumulating evidence that self-compassion is associated with lower levels of fear of recurrence and generalized anxiety among cancer patients. Incorporating such approaches—whether through self-compassion-based interventions or mindfulness-based programs [14,16,48]—may help patients develop greater emotional resilience and more adaptive coping mechanisms in the face of illness-related challenges. In addition, to substantiate the clinical value and effectiveness of these interventions, future research should prioritize longitudinal studies and randomized controlled trials. These designs are essential to establish causal relationships, assess the durability of psychological benefits, and guide the development of evidence-based programs in psycho-oncology.

### 4.3. Limitations

Despite its contributions, this study has several limitations that should guide future research. Its cross-sectional design limits causal inferences, although alternative serial mediation models with reversed mediator orders were not significant, lending some support to the hypothesized direction of mediational effects. However, longitudinal studies are needed to explore potential bidirectional relationships between self-compassion and fear of recurrence and anxiety. This study focused on three main coping strategies (problem-, emotion-, and meaning-focused) as they play crucial roles in managing and overcoming stressful situations in cancer patients. Future research should also consider other strategies, such as avoidant coping, approach coping, adaptive vs. maladaptive coping, or religious coping. Additionally, cancer-specific variables (e.g., type of therapy used, presence of metastases, and co-morbidities) were not included in the present analyses, although they may play a significant role. Nonetheless, previous studies have demonstrated that psychological factors, such as stress management skills, may significantly predict emotional and physical well-being in cancer patients, even when controlling for clinical parameters, including disease stage and comorbidities [49]. Another limitation is the absence of clinical variables (e.g., time since diagnosis, cancer type, disease stage), which may influence the generalizability and interpretation of the findings. Such variables should be included in future studies to allow for a more nuanced understanding of psychological processes in diverse cancer populations. Finally, as we did not specifically measure the level of responsiveness to treatment, future studies should take into account an examination of how patients with different biological/medical responses to treatment vary in their psychological and emotional reactions. Studying responsiveness is especially important in cancer care, where interventions often need to be adapted for factors such as cancer type, stage, and comorbidities.

## 5. Conclusions

Despite these limitations, the present study offers valuable insights and contributes meaningfully to the growing body of research on negative affective states in women with cancer. It provides empirical support for the mediational role of psychological flexibility and coping in the relationship between self-compassion, fear of recurrence, and generalized anxiety. From a theoretical perspective, the findings enhance our understanding of how self-compassion may alleviate fear and anxiety, potentially through greater mental flexibility and improved emotional and meaningful coping—resources previously linked to self-compassionate individuals [2,19] and known to be critical to negative emotions [20], yet not examined in a multiple-mediation model. Practically, the results support the development of interventions targeting self-compassion and psychological flexibility, such as Cognitively Based Compassion Training (CBCT) [10], to reduce fear of recurrence among individuals affected by cancer-related trauma.

## Figures and Tables

**Table 1:** Correlations among self-compassion, psychological flexibility, coping strategies, fear of recurrence, and generalized anxiety.

	*M*	*SD*	1.	2.	3.	4.	5.	6.	7.	8.
1. Positive self-compassion	3.75	0.75	-							
2. Negative self-compassion	3.55	0.72	0.42 ***	-						
3. Self-compassion (total)	3.65	0.87	0.75 ***	0.76 ***	-					
4. Psychological flexibility	4.87	1.00	0.30 ***	0.26 ***	0.34 ***	-				
5. Problem-focused coping	3.51	0.64	0.49 ***	0.57 ***	0.63 ***	0.45 ***	-			
6. Emotion-focused coping	3.29	0.65	0.37 ***	0.39 ***	0.45 ***	0.37 ***	0.43 ***	-		
7. Meaning-focused coping	3.36	0.76	0.59 ***	0.46 ***	0.62 ***	0.52 ***	0.51 ***	0.48 ***	-	
8. Fear of recurrence	2.10	0.65	−0.37 ***	−0.32 ***	−0.41 ***	−0.33 ***	−0.27 ***	−0.38 ***	−0.58 ***	-
9. Generalized anxiety	2.25	0.86	−0.22 ***	−0.13 *	−0.21 ***	−0.43 ***	−0.21 ***	−0.41 ***	−0.44 ***	0.59 ***

* *p* < 0.05; *** *p* < 0.001.

**Table 2:** Mediational effects: bootstrapping standardized estimates and 95% confidence intervals for the final mediational model.

Model Pathways	Estimate	95% CI	
		Lower	Upper
**Model with serial and parallel mediation effects** Positive self-compassion → Psychological flexibility (Mediator 1) → Emotion-oriented coping/Meaning-oriented coping (Mediators 2) → Fear of recurrence	–0.28 ^a^	–0.34	–0.23
Positive self-compassion → Psychological flexibility (Mediator 1) → Emotion-oriented coping/Meaning-oriented coping (Mediators 2) → Generalized anxiety	–0.21 ^a^	–0.28	–0.15
Negative self-compassion → Psychological flexibility (Mediator 1) → Emotion-oriented coping/Meaning-oriented coping (Mediators 2) → Fear of recurrence	–0.17 ^a^	–0.23	–0.11
Negative self-compassion → Psychological flexibility (Mediator 1) → Emotion-oriented coping/Meaning-oriented coping (Mediators 2) → Generalized anxiety	–0.16 ^a^	–0.23	–0.11

^a^ An empirical 95% confidence interval does not overlap with zero.

## Data Availability

The data presented in this study are available at URL: https://osf.io/nrpj4 (accessed on 1 July 2025).
